# Hypnotherapy as an Adjunctive Treatment for Chronic Post-traumatic Stress Disorder With Dissociative Symptoms: A Case Report

**DOI:** 10.7759/cureus.111639

**Published:** 2026-06-28

**Authors:** Lylia Kabeche, Mohamed Nedjari

**Affiliations:** 1 Faculty of Medicine, University of Health Sciences – Dr Youcef El Khatib, Algiers, DZA

**Keywords:** adjunctive therapy, dissociation, hypnosis, post-traumatic stress disorder, ptsd, therapy, treatment

## Abstract

Post-traumatic stress disorder (PTSD) is a mental disorder that develops following exposure to highly stressful, frightening, or distressing events. We present the case of a 54-year-old man who had experienced PTSD symptoms for 27 years and remained untreated until admission to our hospital. He presented with hypervigilance, insomnia, nightmares, flashbacks, and marked irritability. This case report highlights the potential role of hypnotherapy as an adjunctive treatment for PTSD, although it is not considered a first-line intervention.

## Introduction

Many people are exposed to traumatic events during their lifetime, and approximately 70% of the global population will experience at least one such event; however, only 5.6% of individuals subsequently develop post-traumatic stress disorder (PTSD) [1]. Within one year, approximately 40% of affected individuals achieve full recovery. Reported risk factors for PTSD include female sex, early-life trauma, childhood abuse, greater trauma severity, lack of social support, lower educational attainment, race, and increased life stress following trauma exposure [2].

According to the Diagnostic and Statistical Manual of Mental Disorders, Fifth Edition (DSM-5), PTSD symptoms must persist for more than one month and cause clinically significant distress or functional impairment. The main symptom clusters include intrusion symptoms, such as intrusive memories and flashbacks; avoidance symptoms, including avoidance of trauma-related thoughts, memories, feelings, or external reminders; negative alterations in cognition and mood; and alterations in arousal and reactivity, such as hypervigilance and sleep disturbances [3].

Several effective treatments are available for PTSD. Evidence-based psychological interventions remain the preferred first-line approach and may be delivered individually or in groups, either in person or remotely. The American Psychological Association (APA) recommends trauma-focused psychotherapies such as Cognitive Processing Therapy (CPT), Prolonged Exposure (PE), and trauma-focused Cognitive Behavioral Therapy (TF-CBT). Pharmacological treatments are generally considered second-line options and include selective serotonin reuptake inhibitors (SSRIs), such as fluoxetine, paroxetine, and sertraline [4].

Hypnotherapy has been proposed as an adjunctive intervention for PTSD because hypnosis is hypothesized to facilitate focused attention, controlled access to traumatic memories, reduce physiological arousal, and support cognitive and emotional processing of trauma-related experiences. By decreasing avoidance and improving emotional regulation, hypnosis may target key mechanisms involved in the persistence of PTSD symptoms. Although trauma-focused psychotherapies, including CBT and eye movement desensitization and reprocessing, remain first-line treatments, hypnotherapeutic techniques may offer additional benefit in selected patients. Meta-analyses have reported symptom improvement following hypnotic interventions; however, the overall evidence remains limited by the small number of rigorous randomized controlled trials. Accordingly, hypnotherapy is not currently recommended as a first-line treatment in major guidelines, but it may have a complementary role in specific clinical contexts [5,6].

## Case presentation

We report the case of a 54-year-old married man with three children who had experienced PTSD symptoms for 27 years and was hospitalized in a psychiatric unit. He had been in good health until he sustained two gunshot wounds during a terrorist attack, the second of which occurred one year after the first. During the second attack, his friend and colleague was killed at the scene. Subsequently, he developed diabetes mellitus and hypertension and began to exhibit symptoms consistent with PTSD, including anxiety, insomnia, flashbacks, and irritability.

He consulted several psychiatrists over the years and received various pharmacological treatments, including haloperidol and lorazepam, without significant improvement. He continued to experience flashbacks, nightmares, angry outbursts, and dissociative episodes. His condition improved after trauma-focused hypnotherapy was added as an adjunctive treatment, following several years of psychotropic treatment without remission.

At baseline, the patient reported weekly dissociative episodes, most often triggered by trauma-related reminders such as loud slamming doors. These episodes were characterized by derealization lasting approximately 10 minutes and were followed by a brief period of confusion before he returned to his usual anxious and sad state. He also experienced frequent auditory pseudo-hallucinations, occurring several times per day and persisting for several consecutive days.

He recognized these experiences as internally generated and repeatedly expressed distress about them, stating that they had not been present before the traumatic event. The pseudo-hallucinations consisted of an internal voice urging him to commit suicide. He consistently identified these experiences as originating from his own mind rather than from an external source, and no other psychotic symptoms were present.

Given the preserved insight, recognition of the experiences as internally generated, absence of delusions, Positive and Negative Syndrome Scale (PANSS) findings not suggestive of a psychotic disorder [7], and lack of formal thought disorder, these phenomena were considered trauma-related pseudo-hallucinations rather than manifestations of a primary psychotic disorder.

Mental and physical status examination revealed a patient of average height and build who was cooperative and established good rapport. His mood was anxious and sad. Speech was coherent, well-structured, organized, and goal-directed. Thought processes were logical, with no evidence of formal thought disorder. No delusions or impairment in judgment were observed. Cognitive functions, including orientation, attention, memory, and concentration, were intact.

Baseline psychometric assessment demonstrated significant anxiety, with a Hamilton Anxiety Rating Scale (HAM-A) [8] score of 24, and mild depressive symptoms, with a Hamilton Depression Rating Scale (HAM-D) [9] score of five. PTSD symptom severity, assessed using the PTSD Checklist for DSM-5 (PCL-5) [10], was 55, indicating severe symptom burden. Insight was assessed using the Birchwood Insight Scale [11], with a score of 14 indicating preserved insight.

We initiated pharmacological treatment with mirtazapine 30 mg , and amitriptyline oral solution (4%, equivalent to 40 mg/mL) was administered at a dose of approximately 20 drops per day (≈40 mg/day) to reduce anxiety, and risperidone 0.5 mg to reduce pseudo-hallucinations in the absence of evidence of a primary psychotic disorder. In addition to medication, the patient underwent eight hypnotherapy sessions.

During the first two sessions, therapy focused on establishing a safe place and reinforcing the sense of safety associated with it. The patient identified a family-oriented seaside scene as his safe place. Hypnosis was used to induce calm and create this individualized imagery. The purpose of this technique was to improve autonomic calming, emotional regulation, and perceived safety. Repeated use of the safe-place imagery enabled the patient to enter a calm and emotionally secure state more quickly.

In the third session, brief exposure to the trauma was introduced. The patient experienced intense fear and anxiety and came close to dissociation, so he was guided back to his safe place. Under hypnosis, regulated exposure to the traumatic memory was introduced while maintaining emotional distance. When anxiety increased (with marked fear and autonomic activation) or dissociative symptoms appeared (derealization and emotional detachment), he was returned to safe-place imagery to restore emotional stability.

In the fourth, fifth, and sixth sessions, we moved back and forth between the safe place and the trauma. We also asked him to modify his trauma scene by changing colors and decreasing the noise. A dissociative imagery method known as the "cinema screen" was employed from the fourth to the sixth session. The patient established psychological distance from the experience by visualizing the horrific event as though viewing a movie. To improve cognitive control and lessen emotional reactivity, he was urged to alter the sensory elements of the images, such as size, sound, and visual intensity. Every time the level of distress rose, safe-place imagery was invoked.

As treatment progressed, anxiety and fear gradually diminished, accompanied by a progressive reduction in anxiety severity as measured by the Hamilton Anxiety Rating Scale (HAM-A), with scores decreasing from 24 at baseline to 20 at mid-treatment and 12 at the end of therapy (Table 1).

**Table 1:** Clinical and therapeutic progression across the eight hypnotherapy sessions This table summarizes the patient's clinical evolution throughout treatment, including changes in pharmacological management, dissociative symptoms, auditory pseudo-hallucinations, psychometric assessment scores, and overall clinical status. A progressive reduction in anxiety symptoms was observed, with Hamilton Anxiety Rating Scale (HAM-A) scores decreasing from 24 at baseline to 20 at mid-treatment and 12 at the end of therapy. Improvements in dissociative symptoms and auditory pseudo-hallucinations were accompanied by enhanced emotional regulation and overall functioning. HAM-A, Hamilton Anxiety Rating Scale; HAM-D, Hamilton Depression Rating Scale; BIS, Birchwood Insight Scale; PTSD, Post-traumatic stress disorder; CL-5, PTSD Checklist for DSM-5.

Time point	Treatment	Dissociative symptoms	Auditory pseudo-hallucinations	Assessment scales	Clinical observations
Baseline (Session 1)	Mirtazapine + Amitriptyline	Weekly derealization episodes	Several times daily	PCL-5 = 55; HAM-A = 24; HAM-D = 5; BIS = 14	Severe PTSD symptoms
Mid-treatment (Session 4)	Mirtazapine + Amitriptyline + Risperidone 0.5 mg/day	Reduced	Less frequent	HAM-A = 20	Partial improvement
End of treatment (Session 8)	Amitriptyline ± Risperidone*	Resolved	Remitted	HAM-A = 12	Marked clinical improvement
9-month follow-up	Amitriptyline only	Absent	Absent	PCL-5 = 13	Sustained improvement

During the seventh and eighth sessions, future projection was introduced. The patient was asked to imagine himself in the future and to reflect on how he would perceive himself after better regulating his trauma-related emotions. This future-oriented imagery was intended to consolidate therapeutic gains and encourage adaptive self-perception and emotional regulation. The patient was encouraged to use these internal emotional anchors, meaning positive emotional states linked to safety and mastery, when confronted with trauma-related reminders.

By the end of treatment, auditory pseudo-hallucinations had remitted, dissociative symptoms were no longer reported, and the patient demonstrated marked clinical improvement. Risperidone, prescribed at a low dose of 0.5 mg/day as an adjunctive symptomatic treatment for trauma-related pseudo-hallucinations, was progressively discontinued after symptom resolution, while amitriptyline was maintained for residual mild anxiety symptoms.

**Figure 1:**
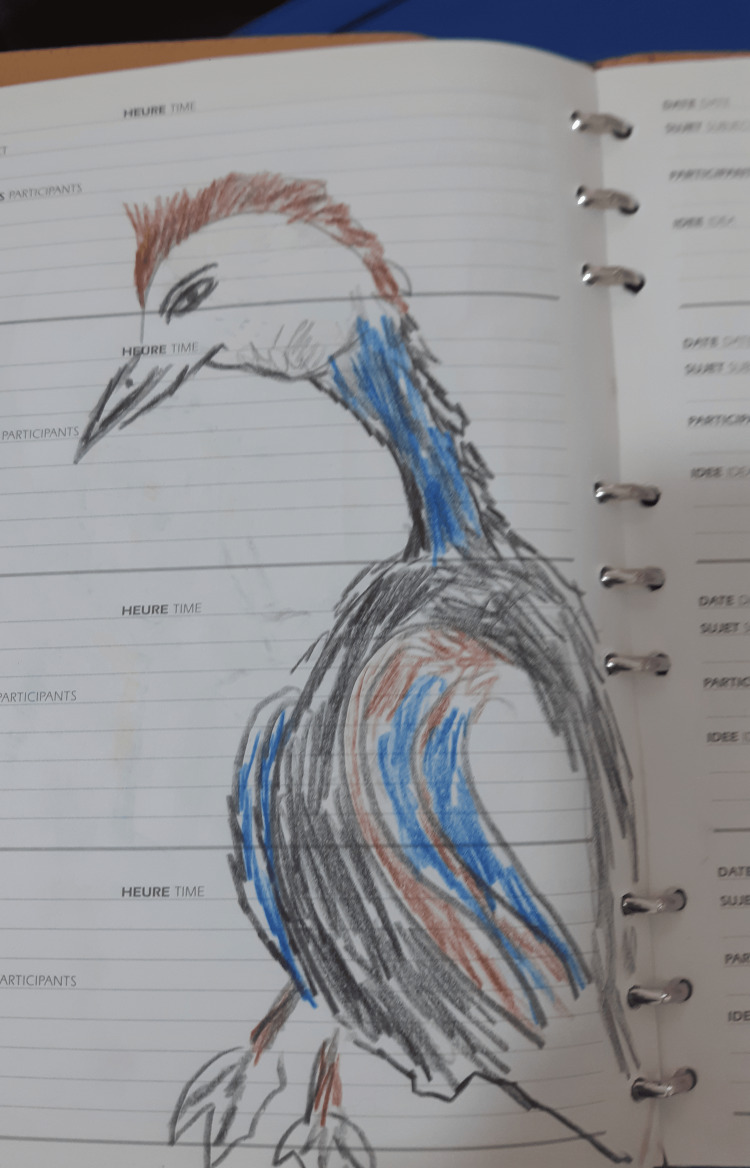
Pre-therapy drawing showing minimal use of color Pre-therapy drawing produced by the patient, characterized by minimal use of color, limited visual detail, and reduced expressive elements. The drawing is presented as a qualitative illustration of the patient's subjective emotional state prior to the initiation of hypnotherapy and was not assessed using a standardized art-therapy evaluation framework.

The drawings were not used as a formal psychometric assessment tool but were produced spontaneously by the patient during the therapeutic process and served as qualitative illustrations of his subjective experience. Figure 1, created prior to hypnotherapy, is characterized by limited use of color, reduced visual detail, and themes reflecting emotional withdrawal and psychological distress.

**Figure 2:**
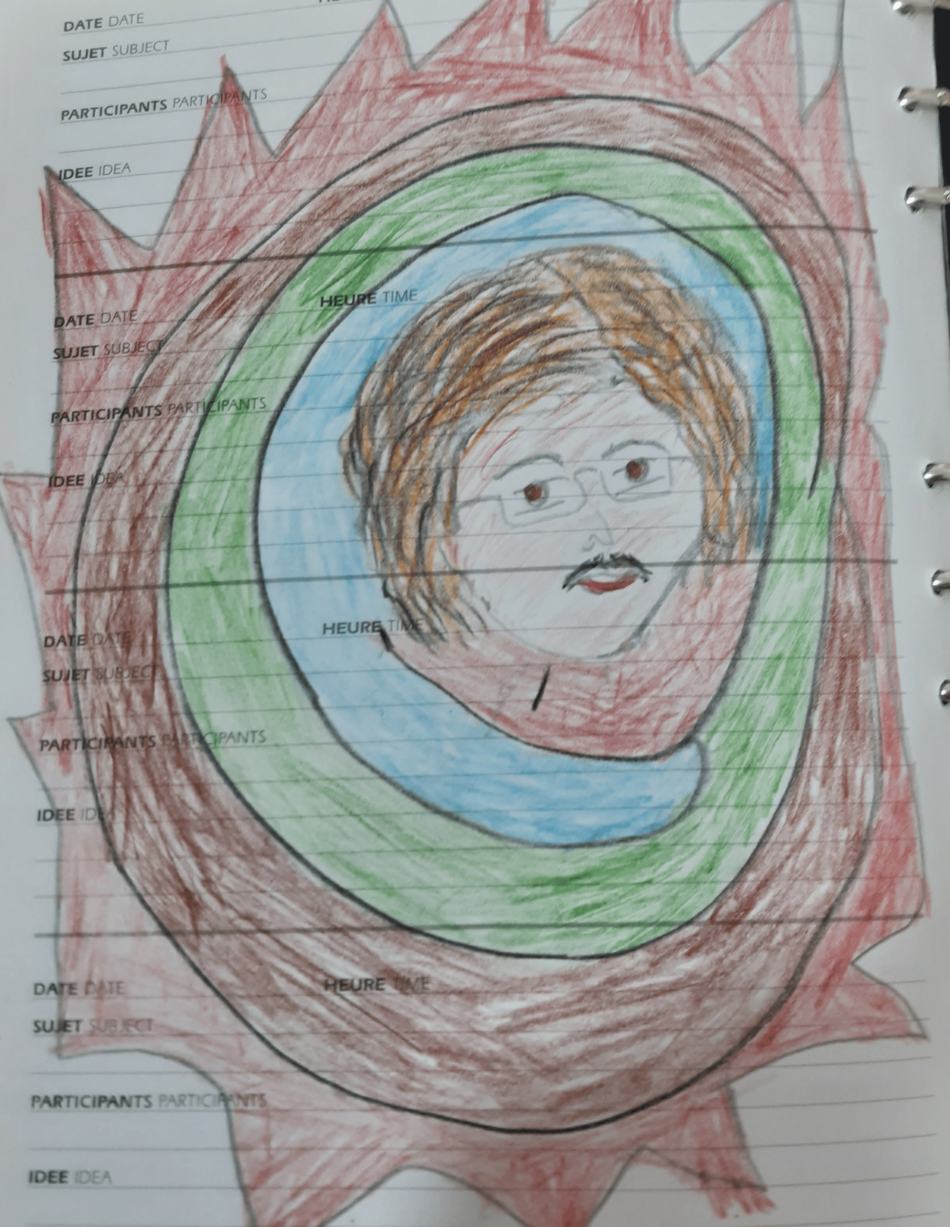
Drawing produced later in therapy, demonstrating increased use of color and visual detail Drawing produced later in the course of hypnotherapy, demonstrating increased use of color, greater visual complexity, and enhanced expressive detail compared with the pre-therapy drawing. These qualitative changes are presented as illustrative of the patient's subjective therapeutic experience and should not be interpreted as objective measures of clinical improvement.

In contrast, Figure 2, produced during the course of treatment, demonstrates greater use of color, increased complexity of visual elements, and a broader emotional range.

These observations were interpreted only as qualitative indicators consistent with the patient's reported clinical improvement and reductions in PTSD symptoms, rather than as objective measures of therapeutic outcome.

Written informed consent was obtained from the patient for publication of this case report and any accompanying images.

## Discussion

The present case suggests a potential beneficial effect of trauma-focused hypnotherapy as an adjunctive intervention for chronic PTSD with dissociative features. Over the course of treatment, the patient demonstrated progressive clinical improvement, including remission of auditory pseudo-hallucinations and dissociative symptoms, reduction in anxiety severity, and substantial improvement in overall PTSD symptomatology. This improvement was reflected by a decrease in the HAM-A score from 24 at baseline to 12 at treatment completion and by a reduction in the PCL-5 score from 55 at baseline to 13 at the nine-month follow-up. These findings are particularly noteworthy given the chronicity of the disorder, as the patient had experienced persistent symptoms for approximately 27 years despite multiple psychiatric consultations and pharmacological interventions.

According to international treatment guidelines, TF-CBT, CPT, PE therapy, and eye movement desensitization and reprocessing are recommended as first-line treatments for PTSD [4].

Although hypnotherapy is not currently recommended as a first-line intervention, it has been proposed as a potentially useful adjunctive approach in selected patients. The theoretical rationale for hypnotherapy in PTSD is based on its capacity to facilitate controlled access to traumatic memories while maintaining emotional regulation and psychological safety. Hypnotic techniques may reduce physiological arousal, enhance attentional control, decrease avoidance behaviors, and facilitate cognitive and emotional processing of trauma-related experiences. These mechanisms may be particularly relevant in patients with dissociative symptoms, for whom direct exposure to traumatic memories can sometimes be difficult to tolerate [12,13].

The therapeutic approach used in the present case incorporated several of these mechanisms. Safe-place imagery was initially employed to establish emotional stability and strengthen the patient's sense of safety before trauma processing was introduced. Subsequent hypnotic exposure allowed traumatic memories to be approached gradually while maintaining emotional distance. The use of imagery modification techniques, including alteration of colors, sounds, and visual intensity, as well as the “cinema screen” method, may have contributed to reducing emotional reactivity and increasing psychological distance from the traumatic event. These interventions are conceptually consistent with contemporary trauma-focused therapies that combine exposure-based techniques with emotional regulation strategies.

Existing evidence regarding hypnotherapy in PTSD remains limited but encouraging. Meta-analytic studies have reported significant reductions in PTSD symptom severity following hypnotherapeutic interventions, suggesting that hypnosis may have therapeutic value as an adjunctive treatment. However, these reviews also emphasized the limited number of available studies and the need for larger, methodologically rigorous randomized controlled trials. Together, these findings suggest that hypnotherapy may contribute to symptom reduction, although its precise role within evidence-based PTSD treatment remains to be clearly established [5].

Our findings are also broadly consistent with previously published case reports involving chronic PTSD and dissociative symptoms. Previous reports have described sustained improvement in PTSD and dissociative symptomatology following structured trauma-focused interventions. Likewise, studies involving patients with the dissociative subtype of PTSD have demonstrated that carefully regulated trauma processing may result in meaningful reductions in both PTSD and dissociative symptoms. Although these interventions differed from hypnotherapy, they share important therapeutic principles with the present case, including gradual exposure to traumatic memories, emotional stabilization, and the reduction of avoidance behaviors. The present observation extends these findings by describing improvement following a brief course of trauma-focused hypnotherapy in a patient presenting not only with dissociative episodes but also with trauma-related auditory pseudo-hallucinations [14].

An additional feature of interest is the remission of auditory pseudo-hallucinations. These experiences were characterized by preserved insight, recognition of their internal origin, absence of delusional beliefs, negative PANSS findings, and the absence of formal thought disorder, supporting their interpretation as trauma-related pseudo-hallucinations rather than manifestations of a primary psychotic disorder. Low-dose risperidone (0.5 mg/day) was prescribed as a symptomatic adjunctive treatment for these symptoms and was subsequently discontinued following their remission. The parallel improvement in pseudo-hallucinations, dissociative symptoms, anxiety, and PTSD symptom severity raises the possibility that successful processing of traumatic memories and enhanced emotional regulation may have contributed to their resolution.

The present case contributes to the existing literature in several respects. First, it illustrates the potential utility of trauma-focused hypnotherapy in a patient with longstanding PTSD that had remained symptomatic for nearly three decades despite multiple treatment attempts. Second, it highlights the possible usefulness of hypnosis in individuals presenting with prominent dissociative symptoms, a subgroup that may experience difficulties engaging in conventional trauma-focused interventions. Third, it documents improvement across multiple clinical domains, including PTSD symptoms, anxiety, dissociative experiences, and trauma-related pseudo-hallucinations, with maintenance of gains at nine-month follow-up. Although conclusions regarding efficacy cannot be drawn from a single observation, the findings support further investigation of hypnotherapy as an adjunctive intervention in chronic and complex presentations of PTSD.

Several limitations should be acknowledged. First, this report describes a single patient and therefore cannot establish a causal relationship between hypnotherapy and clinical improvement. Nonspecific therapeutic factors, including the therapeutic alliance, expectancy effects, and spontaneous symptom fluctuations, may have contributed to the favorable outcome. Second, although psychometric assessments demonstrated substantial improvement, repeated standardized measurements were not obtained at every treatment session. Third, the drawings included in this report were not evaluated using a standardized art-therapy assessment framework and should therefore be interpreted only as qualitative illustrations of the patient's subjective experience. The decision to use hypnotherapy was primarily guided by its accessibility and availability within our clinical setting. Pharmacological treatment was not intended as a stand-alone intervention for PTSD but rather as a supportive measure to alleviate symptoms that could interfere with the therapeutic process, particularly anxiety and sleep disturbances, thereby facilitating engagement in trauma-focused psychotherapy. Finally, hypnotherapy was introduced concurrently with pharmacological treatment, including mirtazapine, amitriptyline, and low-dose risperidone. Consequently, the relative contribution of hypnosis and medication to the observed clinical improvement cannot be determined with certainty. Future controlled studies are needed to clarify the role of hypnotherapy in PTSD, identify the patients most likely to benefit from this approach, and determine its long-term effectiveness when used alongside established evidence-based treatments.

## Conclusions

The present case highlights the potential benefits of trauma-focused hypnotherapy as an adjunctive strategy for persistent, treatment-resistant PTSD. In this patient, hypnotherapy combined with medication was associated with substantial clinical improvement and long-lasting remission of severe symptoms, including dissociation and pseudo-hallucinations, in the absence of evidence of a primary psychotic disorder. These findings suggest that hypnosis may help selected patients tolerate trauma exposure, regulate affect, and process traumatic memories more safely, although evidence-based psychotherapies such as CBT, PE, and eye movement desensitization and reprocessing remain first-line treatments. Further research is needed to clarify the role of hypnotherapy, its indications, and its long-term efficacy.
